# A murine pressure ulcer model for evaluating persistence and treatment of *Staphylococcus aureus* infection

**DOI:** 10.3389/fmed.2025.1561732

**Published:** 2025-04-03

**Authors:** Michele Tavecchio, Silvia Fanni, Xuemin Wu, Ganna Petruk, Manoj Puthia, Artur Schmidtchen

**Affiliations:** ^1^Division of Dermatology and Venereology, Department of Clinical Sciences, Lund University, Lund, Sweden; ^2^Dermatology, Skåne University Hospital, Lund, Sweden

**Keywords:** pressure ulcer, *Staphylococcus aureus*, TCP-25, wound infection, bioimaging

## Abstract

Chronic wounds, particularly pressure ulcers, pose significant healthcare challenges, especially in the elderly population. This study presents an experimental murine model of chronically infected pressure ulcers using a single cycle of magnet-induced ischemic injury combined with infection by bioluminescent *Staphylococcus aureus*. The model addresses previous limitations in studying pressure ulcer infection pathogenesis and evaluating treatment efficacy. By combining this model with *in vivo* imaging system (IVIS) technology, we achieved real-time, non-invasive monitoring of infection dynamics. This approach demonstrated persistent pressure ulcer wound infection and provided temporal and spatial data on infection status. To validate the model’s utility, we evaluated the antimicrobial efficacy of TCP-25, a synthetic host defense peptide, delivered in a topical gel formulation. Our findings highlight the potential of this model for investigating wound infection mechanisms, bacterial persistence, and therapeutic interventions. This innovative approach represents a significant advancement in pressure ulcer research, offering new opportunities for developing effective treatment strategies and improving patient outcomes.

## 1 Introduction

Chronic wounds, including pressure ulcers, venous leg ulcers, and diabetic foot ulcers, mainly affect older adults and pose significant challenges to healthcare systems (1). Pressure ulcers, also known as bedsores or decubitus ulcers, represent a significant medical problem, particularly affecting immobilized or elderly patients. These wounds develop when prolonged pressure on the skin reduces blood flow, leading to tissue damage and necrosis (2). Pressure ulcers are highly susceptible to bacterial colonization and infection, with *Staphylococcus aureus* being one of the most prevalent pathogens isolated from these wounds (3, 4). *S. aureus* infections can significantly impair wound healing and lead to complications, including sepsis, highlighting the need for effective treatment strategies and reliable experimental models.

To better understand the pathogenesis of pressure ulcer infections and evaluate potential treatments, animal models that closely mimic human wound conditions are essential. Mouse models have been widely used in wound healing research due to their cost-effectiveness, ease of handling, and availability of genetically modified strains. Animal models of chronic wounds are limited and challenging to develop (5–7), with few studies demonstrating successful incorporation of exogenous wound pathogens in these models (7, 8). Moreover, developing a reliable murine model of chronically infected pressure ulcers has been challenging due to either self-limiting nature of infection or onset of sepsis (9, 10). This makes the study of pressure ulcer infection pathogenesis and potential treatments difficult.

Recent advances in pressure ulcer modeling have led to the development of more clinically relevant experimental mouse models. One such approach involves the use of magnets to create ischemia–reperfusion injuries that simulate the human conditions leading to pressure ulcer formation (11–13). This method offers several advantages, including controlled and reproducible injury, mimicking human pathophysiology, and being minimally invasive. Use of magnets to induce ischemic injury, presents a more accurate representation of pressure ulcers, enabling the study of complex interactions between ischemia–reperfusion, tissue damage, bacterial colonization, and the host immune response in a controlled setting (11–13).

Wound studies are often invasive and ethically challenging, raising concerns about animal welfare. Non-invasive longitudinal monitoring methods would facilitate unbiased evaluation of wound infection dynamics and treatment efficacy, reducing the need for frequent tissue sampling and animal sacrifice. By using strains engineered to express luciferase enzymes, bacterial growth and dissemination can be tracked in real-time using an *in vivo* imaging system (IVIS). The use of bioluminescent *S. aureus* strains has enhanced our ability to monitor infection progression in various mouse models (14–16). The use of IVIS technology for longitudinal infection imaging offers several significant advantages. It reduces animal usage by allowing repeated measurements on the same animals, enhances statistical power through longitudinal data collection, provides high temporal resolution of infection dynamics, and offers detailed spatial information on bacterial spread. Additionally, bioluminescent signals can be quantified, allowing for objective comparisons between treatment groups and over time. These advantages make the combination of bioluminescent *S. aureus* and IVIS technology a powerful tool for investigating wound infection pathogenesis and evaluating potential therapeutic interventions.

In this study, we have established a pressure ulcer wound mouse model using magnet-induced ischemic injury and infected it with bioluminescent *S. aureus*. The model shows persistent *S. aureus* infection and allows for the evaluation of antimicrobial treatments. We used bioluminescent *S. aureus* and achieved real-time monitoring of infection kinetics and spread. We tested our model to assess the antimicrobial efficacy of a topical gel containing TCP-25, a synthetic host defense peptide that has shown promising antimicrobial activity *in vitro* and *in vivo* (14, 17).

The persistence of *S. aureus* in pressure ulcers is a critical factor with respect to wound healing and infection management. Recent studies have shown that *S. aureus* can persist in pressure ulcers for extended periods, potentially adopting a colonizing role rather than acting as an acute pathogen (18, 19). This persistence may be associated with changes in bacterial virulence and adaptation to the wound environment. Understanding these dynamics is crucial for developing effective treatment strategies and improving patient outcomes.

In conclusion, the development of a pressure ulcer wound mouse model using magnet-induced ischemic injury and infection with bioluminescent *S. aureus* represents a significant advancement for wound research. This model, combined with IVIS technology, offers a unique opportunity to gain insights into the complex interactions between host tissue, bacterial pathogens, and potential therapeutic interventions in a clinically relevant setting. The successful evaluation of TCP-25 gel using this model demonstrates its potential for assessing novel antimicrobial strategies and advancing our understanding of pressure ulcer treatment options.

## 2 Materials and methods

### 2.1 Study design

This study establishes and characterizes a murine model of chronically *S. aureus*-infected pressure ulcers. Balb/c mice underwent a single 16-h cycle of magnet-induced ischemic injury on the dorsal skin to induce pressure ulcers. Following ischemia–reperfusion, wounds were inoculated with bioluminescent *S. aureus* (SAP229). The wounds were then covered in HEC gel (for moisture) and a dressing. Infection dynamics were monitored non-invasively over 14 days using *in vivo* bioluminescent imaging (IVIS) and microbiological analysis (CFU counts from wound swabs and dressings). Wound pathology was assessed via histological analysis and cytokine profiling of wound fluid. Finally, the model’s utility for therapeutic evaluation was demonstrated by assessing the efficacy of a topical TCP-25-containing hydrogel in reducing bacterial burden. A control group, with pressure ulcer without *S. aureus* infection was also studied. Wounds were observed for macroscopic changes, bacterial load, longitudinal infection imaging, and cytokines. Data were analyzed using appropriate statistical methods to compare groups and assess the effects of treatment.

### 2.2 Materials

The peptide TCP-25 (GKYGFYTHVFRLKKWIQKVIDQFGE) was synthesized by Ambiopharm (North Augusta, USA). The purity (95%) of peptide was confirmed by mass spectral analysis (MALDI-ToF Voyager).

### 2.3 Microorganisms

The bacterial strain used for the pressure ulcer wound infection was bioluminescent *S. aureus* SAP229, kindly provided by Dr. Roger D. Plaut (Division of Bacterial, Parasitic, and Allergenic Products, FDA, Bethesda, Maryland, United States). This bacterial strain has been widely used in our experimental wound infection studies and *in vivo* imaging (14–16).

### 2.4 Bacterial inoculation and culture

Bacteria are routinely cultured on Todd Hewitt agar (THA). Using a 1 μL loop, a colony was inoculated in a tube with 5 mL of Todd Hewitt Broth (THB) and cultured overnight at 37°C in a shaking incubator. The following morning, to refresh the culture, 100 μL of the overnight culture was inoculated into a new tube containing 5 mL of THB and incubated at 37°C in a shaking incubator. The bacteria were grown until the optical density (OD) at 620 nm reached between 0.4 and 0.6. The bacteria culture was then centrifuged at 5,600 rpm for 10 min. The supernatant was discarded, and the pellet was washed in 5 mL Tris buffer (10 mM, pH 7.4) and centrifuged again at 5,600 rpm for 5 min. The supernatant was then discarded, and the bacteria were diluted in Tris buffer to a cell density of 2 × 10^9^ CFU/mL. This final bacterial suspension was used to infect mouse pressure ulcer wounds.

### 2.5 Hydrogels used for wound treatments

A hydrogel was prepared using the polymer hydroxyethylcellulose (HEC) (Natrosol™ 250HX, Ashland Inc., UK) and formulated with or without the addition of TCP-25. The gel composition included 1.37% (w/v) Natrosol™ 250HX, 1.21 g/L Tris, and 25 g/L glycerol, adjusted to pH 7.0, with or without 8.6 mg/mL TCP-25.

### 2.6 Murine pressure ulcer model

**Figure 1:**
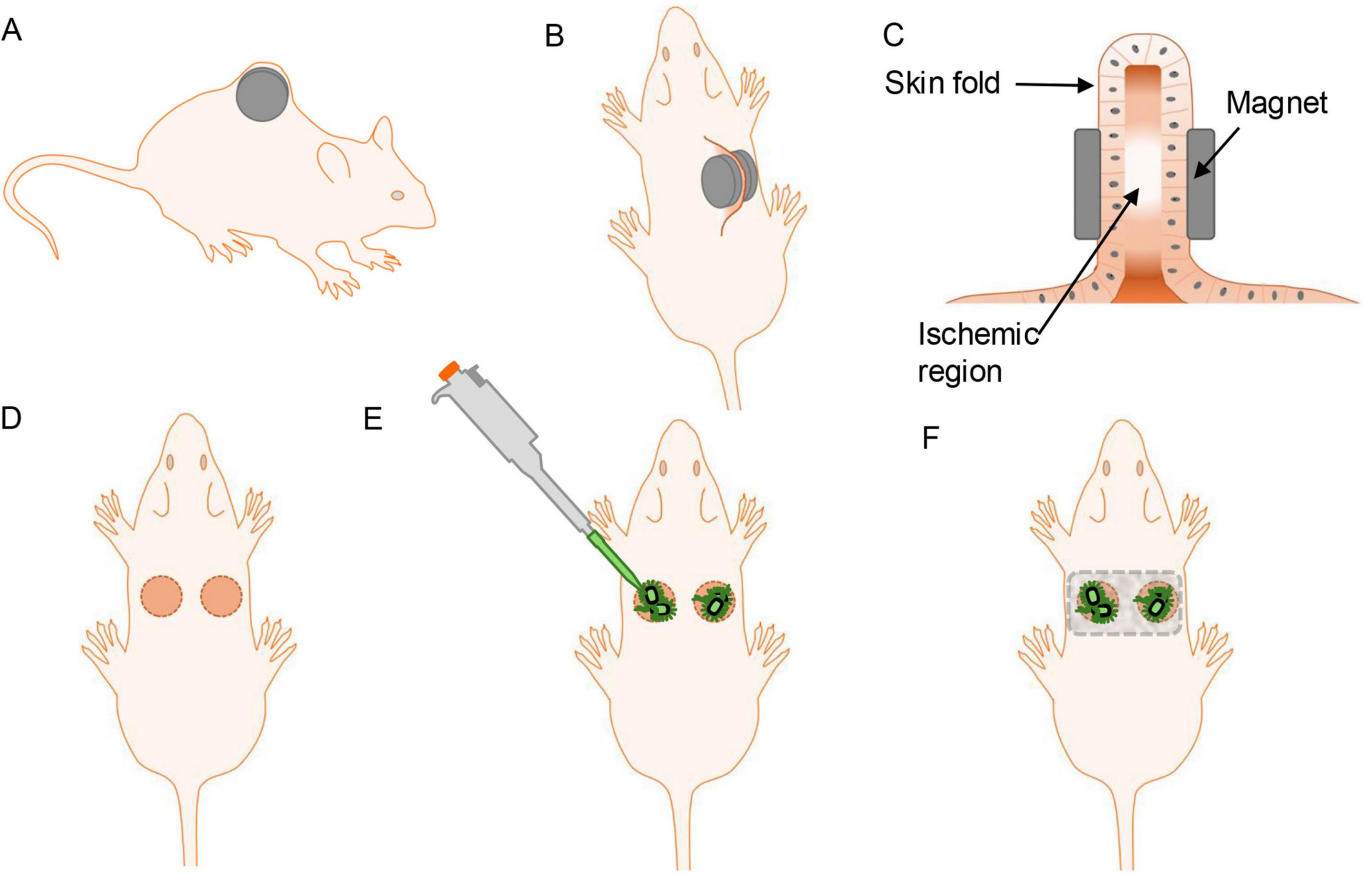
Graphic illustration demonstrating the experimental set-up and workflow of the *S. aureus* infected murine pressure ulcer model. The model employs application of two circular magnets on skin fold on dorsum. **(A,B)** Illustration shows position of magnets on skin fold. **(C)** Pressure exerted by magnets create an ischemic region in the skin fold. **(D)** Upon removal of magnets, two ischemic wound regions can be observed on dorsal skin. **(E)** The wounds are inoculated with an *S. aureus* bacterial suspension. **(F)** Wound dressings are applied to protect the wounds.

**Figure 2:**
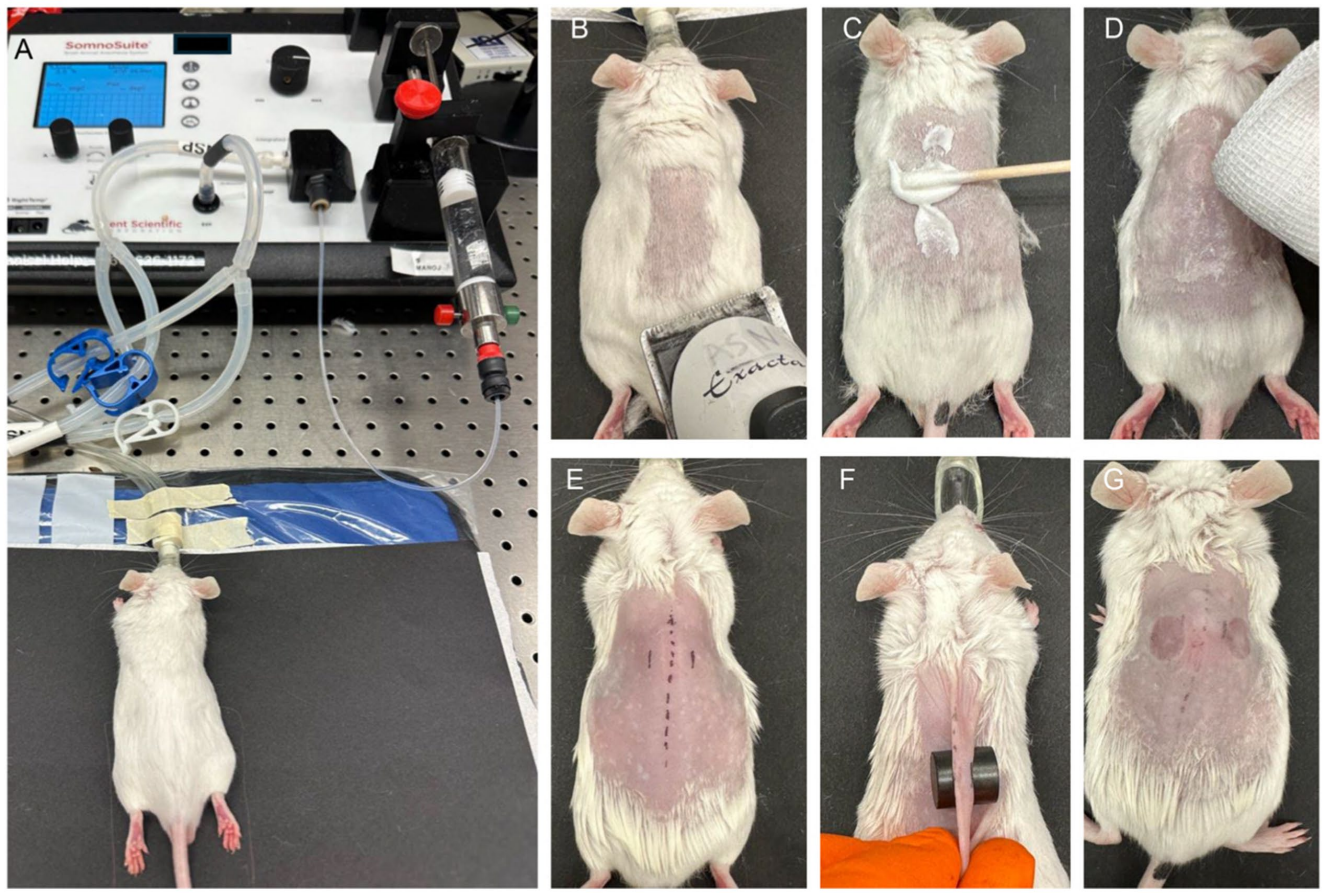
Images showing murine pressure ulcer model during the various procedural steps. **(A)** Mouse is anaesthetized with isoflurane using a Low-Flow Anesthesia system. **(B)** Hair from the back is trimmed using a hair-trimmer. **(C)** Hair depilation is achieved using a depilatory cream. **(D)** Depilatory cream is removed, and the skin cleaned. **(E)** Using a skin marker, area is marked for positioning magnets. **(F)** Two magnets are placed on the skin fold. **(G)** After desired time, magnets are removed. Two ischemic regions can be seen after removal of magnets.

Graphic illustrations and photographs from the experimental set-up and workflow of the *S. aureus* infected murine pressure ulcer model are presented in Figures 1, 2. Balb/c mice (8-12-week-old male; Janvier Labs, France) were used for the study. In a low-flow anesthesia system (SomnoSuite, Kent Scientific), mice were anaesthetized using isoflurane (Baxter; 4% for induction and 2% for maintenance). All procedures were performed under aseptic conditions. Hair from the dorsum were removed using a hair trimmer. For depilation, using a cotton swab, a depilatory cream was applied on the trimmed area and 2 min later, the area was cleaned with a gauze. Finally, to remove any traces of cream, the area was cleaned with prewet gauze and dried. Using a skin marker, midline and area of magnet placement were marked. Approximately 1 cm space was left in between two magnets. Pressure ulcers were induced by sandwiching the skin between two round ferrite magnets (12 × 5 mm; MAGNORDIC, Denmark), each with a pulling force of 0.3 kilogram. The mice were immediately moved to their cages. Mice with magnets showed normal behaviors and no signs of discomfort was observed. After 16 h, the magnets were removed, and the animals were allowed to rest for 6 h to facilitate skin reperfusion. Clear, round ischemic areas were observed at the place where magnets had been applied. Subsequently, the ischemic wounds were infected with 10^4^ CFU of *S. aureus* suspension (10 μL). In a group of animals, control wounds were not infected with bacteria. To maintain moisture, 50 μL of HEC gel was applied to the wounds, followed by covering with a layer of primary dressing (Mepilex transfer; Mölnlycke Health Care) and a secondary dressing (Tegaderm film, 3 M). Finally, a layer of flexible self-adhesive bandage (Vet Flex, Kruuse, Denmark) was applied over the dressings and around the body. Under isoflurane anesthesia, dressing changes were performed on days 1, 2, 4, 6, 8, 10, and 12. On each dressing change, photography, wound swab sample collection, IVIS imaging was done, and new HEC gel was applied. The experiment was terminated on day 14, and wound skin tissue was collected in 10% neutral buffered formalin for further histological analysis. Wound dressing weights and pictures, as well as wound pictures, were recorded daily.

For the therapeutic evaluation of TCP-25 gel, treatments were initiated 30 min after bacterial inoculation. HEC gel (vehicle) or 8.6 mg/mL TCP-25 gel (50 μL) was applied to the wounds, followed application of dressings as described above. Dressing changes were performed on days 1, 2, 4, and 6. On each dressing change, photography, wound swab sample collection, IVIS imaging was performed, and new control gel or TCP-25 gel was applied. The selection of 8.6 mg/mL TCP-25 concentration was motivated by our previous study (14).

### 2.7 Wound photography and area analysis

A DSLR camera (Canon EOS Rebel T7i, Japan) equipped with a 60 mm f/2.8 macro lens (Canon EF-S 60 mm, Japan) was used to capture high resolution photographs of the pressure ulcer wounds. A ring flash system (Canfield Twin Flash, United States) was used to ensure standardized lighting (20). A photography copy stand (Kaiser RS 2 XA) with a baseboard was used to keep standard camera distance and angle conditions. Wound photographs were taken immediately after dressing removal. A disposable single-use sticky ruler was attached on the mouse back below the wound area. Wounds pictures were taken including the ruler, to use it as reference during analyses with ImageJ (National Institute of Health). Upon opening an image with the program, the 1 cm distance represented by the ruler was converted to pixels by mean of the “Straight” command. Briefly, the two lines 1 cm far on the ruler, were connected by the “Straight line” command. The length of the generated straight line, representing 1 cm, was converted in pixel by the “Analyze”== > “Set Scale” command, setting “Known distance” as 1 and cm as “Unit of Length.” Then, each wound perimeter was manually followed using the “Freehand” command. The area included in the traced perimeter was then measured in mm^2^ by the “Analyze”== > “Set Scale” command. To have a precise measure for each wound, the procedures were repeated for every image.

### 2.8 Bacterial CFU analysis

Dressings and swabs from wounds were soaked in 500 μL of PBS and vortexed to release bacteria. Seven serial dilutions were performed, and the samples were plated on TH agar plates and incubated for 16 h at 37°C in a 5% humidified CO_2_ incubator. Then, the colonies were counted, and CFUs calculated.

### 2.9 Cytokine assay

For cytokine analysis, wound fluid was extracted from the primary dressing (Mepilex Transfer). Dressings from wounds were soaked in 500 μL of PBS in 1.5 mL tube and vortexed. The tubes were then centrifuged (600 × *g* at 4°C, 5 min), and the supernatant was collected for cytokine measurement. Cytokines were measured using the CBA Mouse Inflammation Kit (Becton Dickinson) according to the manufacturer’s instructions. In brief, the kit utilizes beads with different fluorescence intensities, each coated with antibodies against specific murine cytokines that can be quantified simultaneously after the addition of a PE-conjugated secondary antibody to the reaction mix.

### 2.10 IVIS imaging

Pressure ulcer wound infection was longitudinally evaluated by measuring bacterial bioluminescence using non-invasive imaging. An *In vivo* imaging system (IVIS spectrum, Perkin Elmer) coupled with Living Image 4.5.5 Software (PerkinElmer) was used. IVIS imaging was performed as described previously (21). In brief, mice were anesthetized in induction chamber using 4% isoflurane-mixed oxygen. Dressings were removed and mice were then transferred to the IVIS imaging chamber and anesthesia was maintained using 2% isoflurane. The mice were positioned in prone position. In Living Image software’s IVIS acquisition control panel, luminescent imaging mode and auto exposure was used for imaging. After image acquisition, a region of interest (ROI) was selected around the wound and the bioluminescence was measured using Living Image program. Heatmaps were created to visualize signals in produced images.

### 2.11 Histology

Skin tissue harvested from the pressure ulcer wounds was placed on absorbent paper to prevent curling and fixed overnight in 10% neutral buffered formalin. After serial dehydration, the tissue was embedded in paraffin blocks, sectioned at 5 μm, and stained with hematoxylin and eosin (H&E). Samples were imaged with bright field microscopy (Axioplan2, Zeiss, Germany) under 100× and 200× magnifications. From each H&E-stained sections, 4–5 microscopic views (100×) were scored which covered most of the wound area. Scoring was done on a scale of 0–5 (where 0 is worse and 5 is best score) (14). The histology scoring was based on epithelization, granulation tissue, inflammatory cells, abscesses and tissue architecture.

### 2.12 Data analyses

Differences between groups were statistically analyzed using Student’s *t*-test for normally distributed data and the Mann–Whitney U test for non-normally distributed data. For grouped analysis, means of more than two groups were compared using two-way ANOVA followed by Sidak’s multiple comparison test. Data are presented as means ± SEM. Details of statistical analysis are indicated in each figure legend and GraphPad Prism software v10 was used. *P*-values <0.05 were statistically significant.

## 3 Results

### 3.1 Characteristics of *S. aureus* infected murine pressure ulcer model

This model utilizes application of two circular magnets over a skin fold, followed by inoculation of the wound area with bioluminescent *S. aureus* (Figures 1, 2). Briefly, mice were anaesthetized, and dorsal skin area was prepared by depilation and cleaning. To induce ischemia, the dorsal skin fold was compressed between two circular magnets for 16 h. After magnet removal, the ischemic wound area was infected with luminescent *S. aureus* and a neutral hydroxyethyl cellulose (HEC) gel was applied to keep the wound area moist. Wounds were covered with a primary polyurethane (PU) foam dressing and secured with Tegaderm film dressing. Subsequently, the wounds were analyzed for macroscopic changes, bacterial load, longitudinal infection imaging, and cytokines.

**Figure 3:**
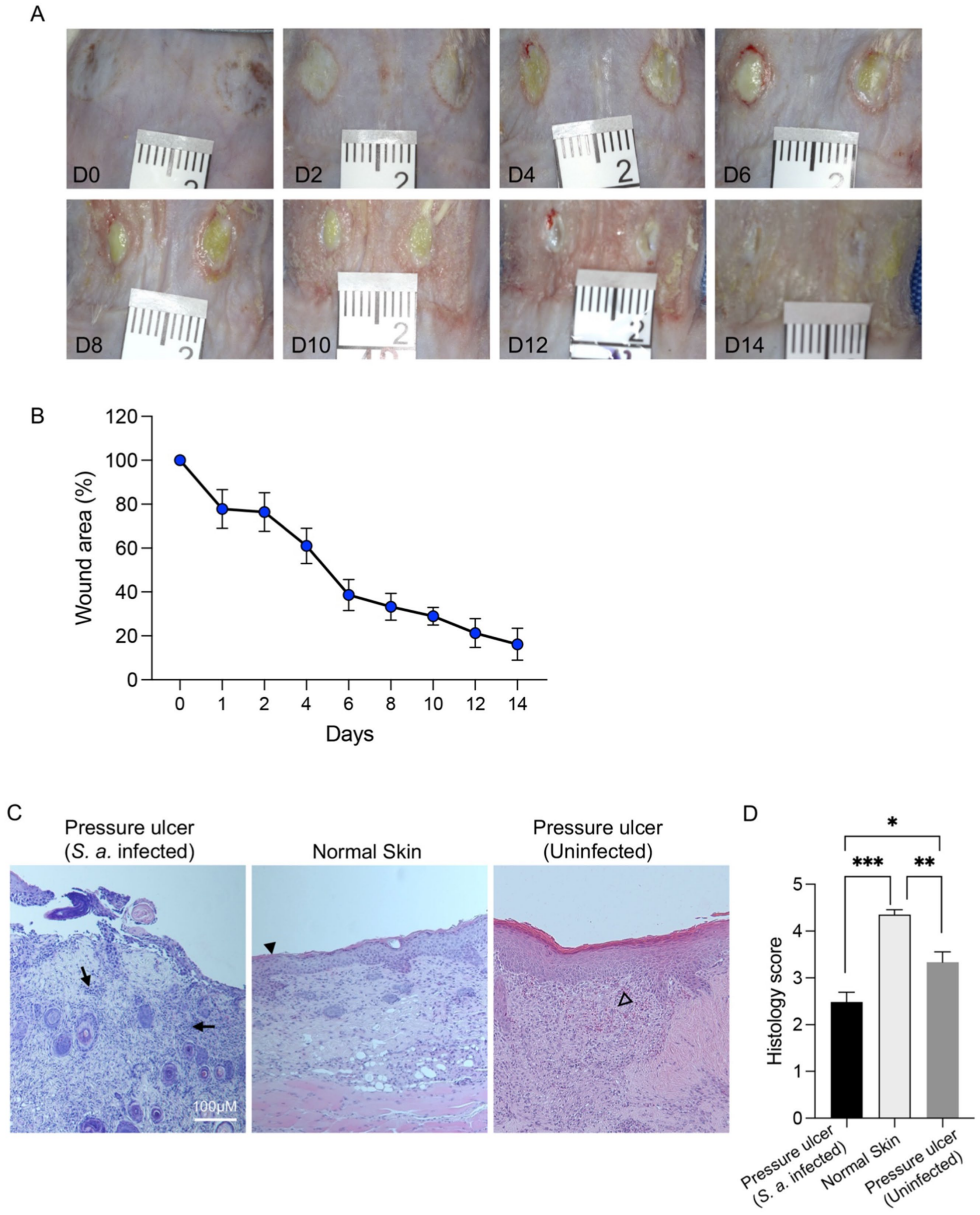
Characteristics of wound infection and tissue pathology in *S. aureus* infected murine pressure ulcer model. **(A)** Photographs show macroscopic appearance of pressure ulcer wounds during 14 days of experimental protocol. **(B)** The wound area was measured and represented here as percent wound area. **(C)** H&E staining was used to study wound tissue histology. **(D)** Bar-chart shows histology scoring. Uninfected pressure ulcer wounds and normal unwounded skin was used for the comparison. Data are represented as the mean ± SEM (*n* = 6). *P*-values were determined using an unpaired *t*-test. Significant results are denoted as ***p < 0.001. Arrow, immune cell infiltration; filled arrowhead, epidermis; hollow arrowhead, distinct area of proliferative phase characterized by high cellular activity.

One ischemia–reperfusion cycle using a single application of magnets for 16 h was sufficient to produce visible circular ischemic regions on the skin (Figure 3A). After removal of magnets at day 0, pale circular wounds were clearly observed. Within pale areas, some petechiae can be observed which might be due to the subcutaneous hemorrhage due to the pressure exerted by the magnets. At day 0, the wounds were infected with *S. aureus*, a common pressure ulcer wound pathogen (3, 22). At day 2, the pale ischemic wound area started to appear yellowish as a sign of infection establishment. Wounds remained yellowish until day 10 and purulent discharge could be seen upon dressing changes. In this model, as expected, severe contraction was observed as wound size reduced over time and reached to almost 25% of the initial wound area at day 14 (Figure 3B). In contrast to the *S. aureus*-infected wounds, the control group with non-infected pressure ulcers showed no purulent discharge (Supplementary Figure 1A). However, the degree of wound contraction observed in the non-infected control group was somewhat similar to that of the infected wounds (Supplementary Figure 1B). This suggests that the wound contraction may be primarily driven by the initial ischemic injury and subsequent healing processes, rather than being solely dependent on the presence of infection.

Hematoxylin and eosin (H&E) staining of wound biopsies collected at day 14 showed significant tissue changes. Compared to the normal unwounded skin, no visible epidermis, no distinct dermis and basement membrane were observed in the pressure ulcer wounds (Figure 3C). Skin tissue was significantly infiltrated with immune cells and normal skin tissue architecture was completely absent. Histology scoring showed significantly poorer score for the pressure ulcer wound tissue (Figure 3D). The non-infected pressure ulcer wounds exhibited distinct areas of proliferative phase characterized by high cellular activity and re-epithelialization. While the skin tissue architecture in non-infected wounds was significantly better than in infected wounds, it still showed impairment compared to normal skin tissue. Notably, fewer inflammatory cells were observed in non-infected wounds compared to their infected counterparts. The histology score of non-infected wounds was significantly better than infected wounds but poorer than normal tissue, providing a clear demonstration of the impact of bacterial infection on wound healing and tissue integrity. Taken together, results show that this experimental method produces reproducible pressure ulcer wounds with significant tissue changes over a 14-day period.

### 3.2 Microbiological analysis and persistence of infection

**Figure 4:**
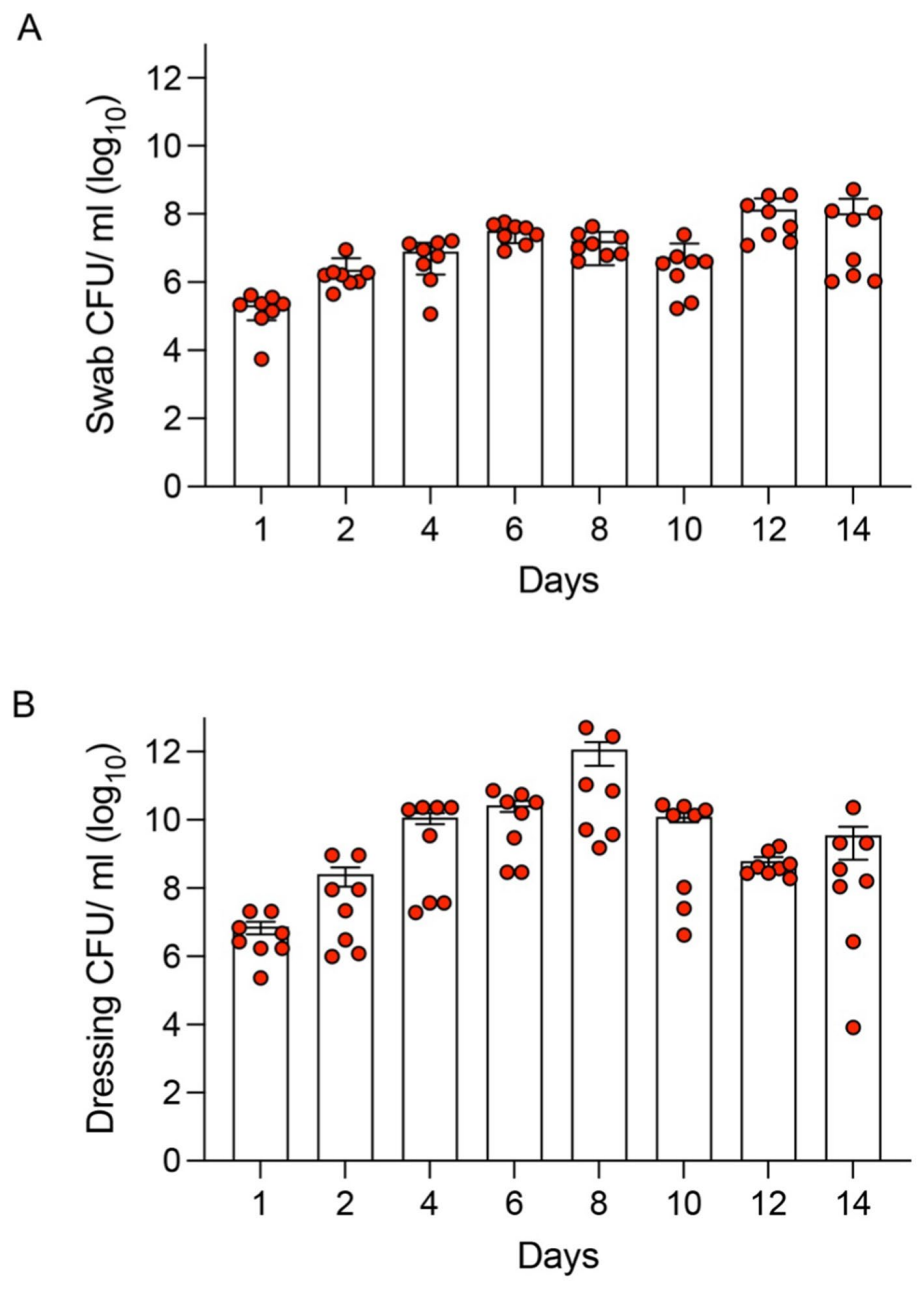
Colony Forming Unit (CFU) analysis of *S. aureus* infected murine pressure ulcer wounds. **(A)** Wound swab samples were taken at days 1, 2, 4, 6, 8, 10, 12, and 14 and CFUs were determined. **(B)** In addition, wound dressings were collected and analyzed for CFU. Data are presented as the mean ± SEM (*n* = 8). Red data points overlaid on the bar chart represent the individual Colony Forming Unit (CFU) counts obtained from each wound.

We next investigated whether *S. aureus* could establish a persistent infection within the pressure ulcer wounds created by our model. Ischemic wounds were infected with a low inoculum of *S. aureus* (10^4^ CFUs). Wound dressings were changed at days 1, 2, 4, 6, 8, 10, 12, and 14. During dressing changes, wound swab samples were taken, and primary dressing material was collected for microbiological analysis. A significant wound bacterial load was observed over the 14-day study period (Figure 4A). Wound swab CFU counts showed an increasing trend until day 8 after which it appeared to be stable until day 14. The CFU count from the dressings was higher than the swab CFU count but showed a similar trend (Figure 4B). No mice showed signs of sepsis or mortality during this period. The results showed a persistent infection in these pressure ulcer wounds.

### 3.3 Longitudinal *in vivo* imaging of infection and cytokine analysis

Longitudinal in vivo imaging was conducted to track infection kinetics and verify that the persistent infection was specifically due to *S. aureus*. Bioluminescent bacterial strains allow monitoring of bacterial growth and spread in real-time through bioimaging with an *in vivo* imaging system (IVIS), facilitating non-invasive longitudinal studies of infection dynamics. This approach offers an advantage, as microbiological analysis of wounds with agar-based methods do not rule out the possibility of wound contamination by other host bacteria from the mice. Therefore, we used luminescent *S. aureus* in combination with IVIS imaging.

**Figure 5:**
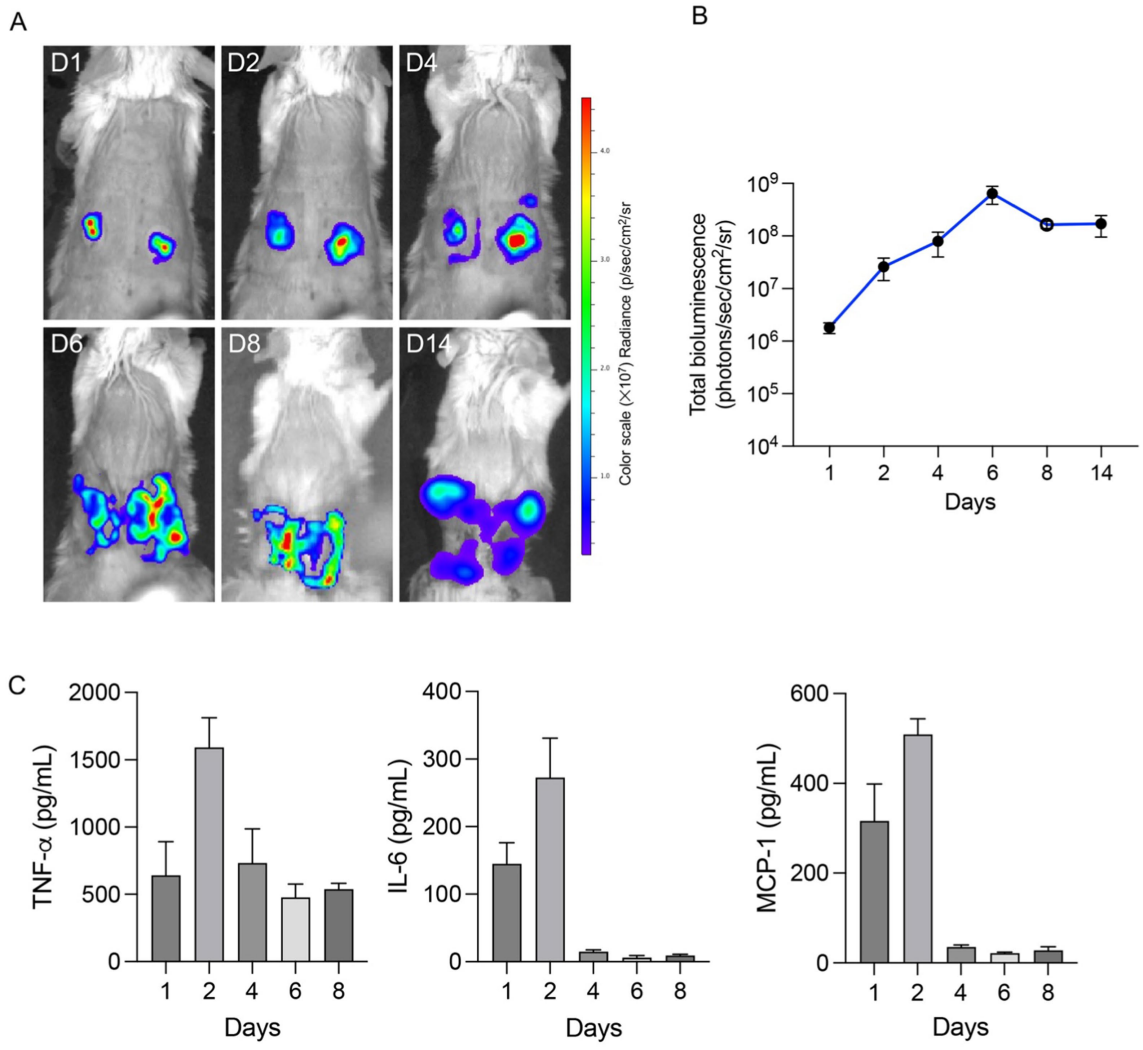
Longitudinal *in vivo* infection imaging and cytokine analysis in the mouse model of *S. aureus* infected murine pressure ulcer wounds. **(A)** To achieve imaging of bacterial infection, pressure ulcer wounds were infected with bioluminescent *S. aureus* bacteria and were non-invasively analyzed using the IVIS bioimaging system. Representative heat-map images show bacterial luminescence at days 1, 2, 4, 6, 8, and 14. **(B)** The line graph shows measured bioluminescence intensity emitted by the bacteria. Data are presented as the mean ± SEM (*n* = 8). **(C)** Analysis of wound fluid cytokines collected on days 1, 2, 4, 6 and 8. Data are presented as the mean ± SEM (*n* = 6).

Mice were imaged at days 1, 2, 4, 6, 8, and 14. In agreement with the CFU data, the pressure ulcer wounds showed significant infection with *S. aureus* which increased until day 6 and then appeared to stabilize (Figures 5A,B). Superficially, infection appeared to spread outside the wound margins, however most of the bacterial load appeared to be within the pressure ulcer wounds. As evidenced by the intense bioluminescent signal, *S. aureus* was localized within the pressure ulcer wounds, and no luminescent emission was observed from other parts of the body indicating that the experimental model did not lead to bacterial spread and sepsis. In conclusion, the results showed that persistent infection is indeed due to *S. aureus* and moreover, that the infection is limited to the pressure ulcer wound area and surroundings.

We next examined whether tissue changes in the pressure ulcers were associated with altered levels of pro-inflammatory cytokines in the wound fluid. Cytokines were measured in wound fluid extracted from dressing materials on days 1, 2, 4, 6 and 8. TNF-*α*, IL-6 and MCP-1 were detected, with notably higher levels observed during the initial days (Figure 5C).

### 3.4 Therapeutic evaluation of a TCP-25 containing hydrogel

**Figure 6:**
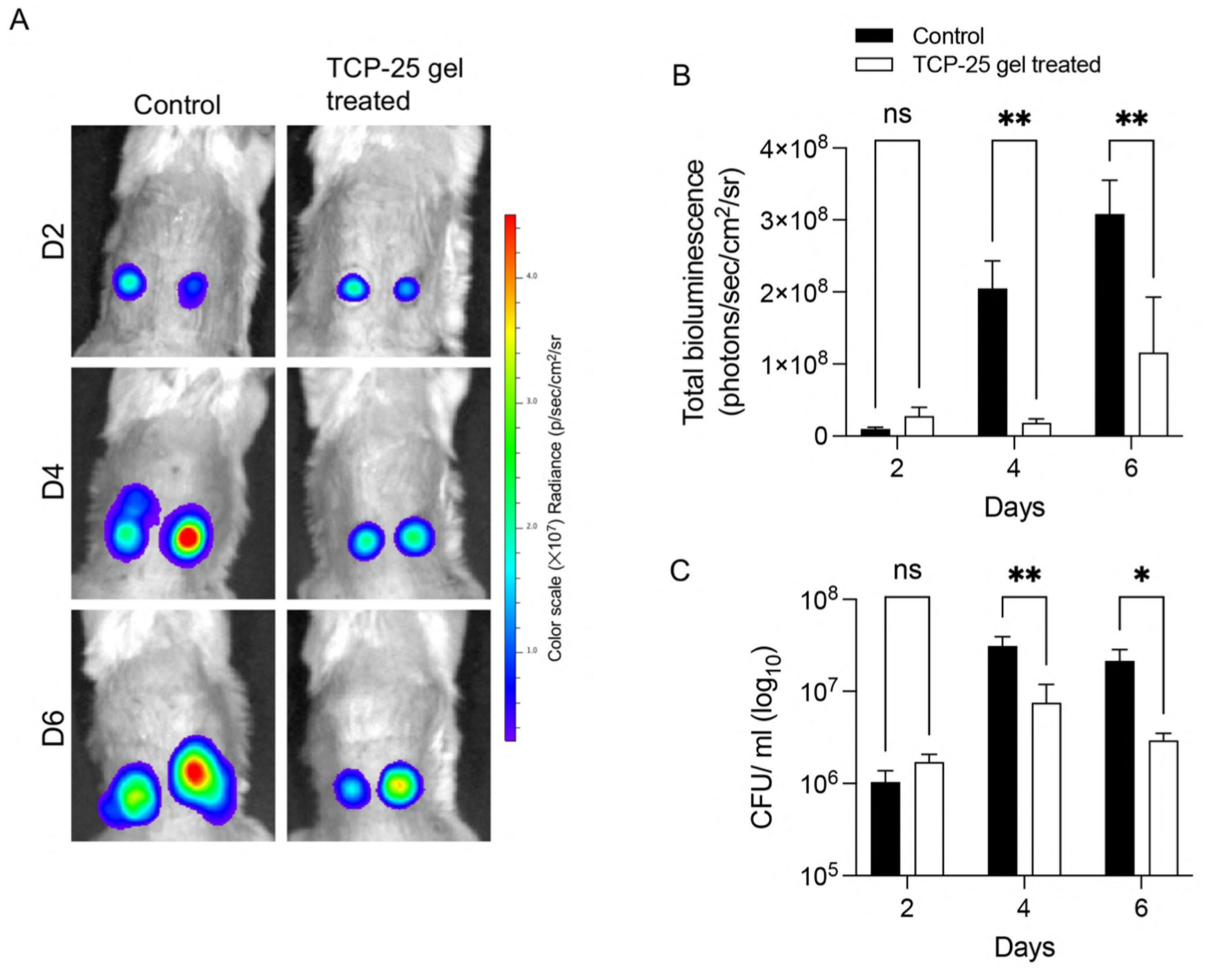
Evaluation of therapeutic effects of TCP-25, a host defense peptide, in *S. aureus* infected murine pressure ulcer wounds. *S. aureus* infected murine pressure ulcer wounds were treated daily with a TCP-25-containing hydrogel. Control wounds were treated with only hydrogel. **(A)** Longitudinal *in vivo* infection imaging using IVIS. Representative heat-map images show bacterial luminescence at days 2, 4, and 6. **(B)** The bar-chart shows measured bioluminescence intensity emitted by the bacteria. Data are presented as the mean ± SEM (*n* = 6). **(C)** Microbiological analysis of TCP-25 gel treated pressure ulcer wounds. Wound swab samples were taken at days 2, 4, and 6 and CFUs were determined. Data are presented as the mean ± SEM (*n* = 6). *P*-values were determined using a two-way ANOVA. *p ≤ 0.05; **p ≤ 0.01; ns, not significant.

A disease model’s utility depends on its ability to accurately differentiate therapeutic responses, thereby bridging the gap between preclinical research and clinical outcomes. Therefore, we wanted to test if the here described *S. aureus* infected pressure ulcer model can be utilized for treatment evaluation. We have previously shown that a host defense peptide, TCP-25, formulated in a hydrogel is effective against *S. aureus* wound infection in a porcine partial thickness wound model (14). In a proof-of-concept study, *S. aureus* infected pressure ulcer wounds were treated with 8.6 mg/mL TCP-25 gel. Wound dressings were changed on days 1, 2, 3, 4, and 6 and new gel was applied. An identical gel without TCP-25 was used as control. Non-invasive IVIS imaging was performed on days 2, 4 and 6. Results showed a significant reduction in bacterial infection in the wounds treated with TCP-25 gel (Figures 6A,B). Reduced bacterial spread was also observed in the TCP-25-gel treated wounds. Consistent with the IVIS imaging results, swab CFU count showed a significant reduction in CFUs in the wounds treated with TCP-25 gel (Figure 6C). Wound photographs show reduced wound exudate in TCP-25 treated wounds, suggesting a reduction in inflammation and bacterial load, and a cleaner, less inflamed appearance compared to control wounds, although macroscopic differences are subtle (Supplementary Figure 2). Taken together, these findings show that the *S. aureus* infected pressure ulcer model presented in this work can be used to evaluate efficacy of antimicrobial therapies.

## 4 Discussion

Various published models vary in their approach in mimicking the clinical conditions leading to pressure ulcers, with some focusing on ischemia–reperfusion injury, others on continuous pressure, and some combining multiple factors like Spinal cord injury (SCI). The choice of model depends on the specific research questions and the aspects of pressure ulcer pathophysiology being studied. Stadler et al. developed a model using two magnets to sandwich a pinch of skin, creating ischemia–reperfusion cycles by removing and reapplying the magnets (11). Similar approach was used by other studies where two round ceramic magnetic plates generating 50 mmHg compressive pressure and three 12-h ischemia/12-h reperfusion cycles were applied (11, 12, 23, 24). A model using a modified compression device delivering 150 mmHg pressure to human full-thickness skin grafts on mice was also developed (25). Three cycles of 8-h compression and 16-h release are applied. Another study reported a model involving implantation of one magnet under the skin and placing another externally, sandwiching the skin for 7 consecutive days (26). A model combining complete spinal cord transection with magnetic disc compression of a skin fold for 12 h, mimicking pressure ulcers in SCI patients has also been described (27). However, these studies did not address establishment of infection. Most clinical cases of pressure ulcer show presence of one or more bacterial species. Hence, in this study, focus was to establish a chronically infected pressure ulcer wound model so that dynamics and pathophysiology of infection and treatment efficacy could be studied.

Our approach, using magnet-induced ischemic injury combined with bioluminescent *S. aureus* successfully addresses previous limitations in modeling pressure ulcer infections. Most other studies have used more than one cycles of ischemia reperfusion (11, 13). However, the model described here uses a single ischemia perfusion cycle, minimizing animal discomfort and reducing time requirements.

Infection establishment requires ambient and favorable conditions for bacterial colonization, and they are difficult to create specially when a single species of bacteria is exogenously inoculated to the wound. Among others, one important factor is maintenance of moisture at the site of inoculation. Using HEC gel to keep wounds moist, we have previously shown successful bacterial colonization of porcine partial thickness wounds (14). In the mouse model, pressure ulcers wounds are not open and comparatively dry during first few days. After bacterial inoculation and at each dressing change, we used HEC gel which promoted bacterial colonization despite a low inoculum size. The bacterial numbers increased during the first week and then stabilized. The sustained bacterial colonization observed over 14 days without causing sepsis closely mimics clinical pressure ulcer infections. The use of bioluminescent *S. aureus* with IVIS imaging enabled real-time tracking of infection dynamics, providing detailed spatial and temporal information about bacterial spread and treatment efficacy. In addition, this approach confirmed that bacterial colonization observed in imaging is indeed due to *S. aureus*. The IVIS system specifically detects light emitted by engineered *S. aureus* strain, providing real-time monitoring of infection dynamics. The localized and intense IVIS signals within the wounds strongly indicate that *S. aureus* is the predominant, and likely causative, agent of infection. While we cannot rule out the presence of other host bacteria at low levels, their contribution to the overall infection is likely minor given the IVIS findings. The persistent presence of *S. aureus* in pressure ulcer wounds points to a chronic infection that likely disrupts normal wound healing processes through sustained inflammation and tissue damage.

The histological changes and proinflammatory cytokine profiles observed in our model reflect the complex pathophysiology of human pressure ulcers. The presence of TNF-*α*, IL-6, and MCP-1 in wound fluid, particularly elevated in the early phase, indicates an active inflammatory response typical of chronic wounds. The use of wound fluid extracted from the primary dressing material enabled us to perform longitudinal cytokine analysis non-invasively. Wound histology at 14 days showed tissue necrosis, abnormal tissue architecture, severe inflammation and immune cell infiltration.

The utility of an animal model is determined by its ability to not only address fundamental biological research questions but also to effectively evaluate the efficacy of potential therapeutic interventions. We selected TCP-25 due to its unique combination of antimicrobial and anti-inflammatory properties, making it an ideal candidate for wound treatment. This thrombin-derived peptide demonstrates effective antibacterial activity against both Gram-positive and Gram-negative bacteria, including *S. aureus* and *P. aeruginosa* (14). Importantly, TCP-25 also scavenges pathogen-associated molecular patterns (PAMPs) like lipopolysaccharide (LPS), preventing CD14 interaction and Toll-like receptor dimerization, thus reducing downstream immune activation (28). This dual-action mechanism allows TCP-25 to address both infection and inflammation simultaneously. Its usability is demonstrated by its ability to be incorporated into hydrogel formulations (14) and dressings (16), allowing for various application methods in wound treatment. TCP-25 has shown therapeutic potential in experimental models of bacterial sepsis, endotoxin shock, and wound infections, and exhibits broad-spectrum activity against multiple bacterial species while modulating responses to various microbe-derived agonists (14, 15, 17). These comprehensive attributes make TCP-25 a promising candidate for developing novel wound treatments that effectively target both bacterial infection and inflammation. Our successful evaluation of TCP-25 gel demonstrates the model’s utility for testing novel antimicrobial formulation. The ability to quantitatively assess treatment outcomes through both IVIS imaging and traditional microbiological methods provides robust validation capabilities. While IVIS offers the distinct advantages of specific pathogen detection, repeated measurements on the same animal, high temporal resolution, and detailed spatial information, alternative methods can indeed be used to assess *S. aureus* infection in our model. For example, selective media like mannitol salt agar can effectively isolate *S. aureus*. Furthermore, 16S rRNA gene sequencing provides a comprehensive assessment of the wound microbiome, while qPCR offers a means of quantifying *S. aureus* DNA. We also recently demonstrated a simple filter paper-based method for spatial analysis of wound infection (20). There are several limitations in our study. First, our model focuses on a single bacterial species (*S. aureus*), whereas clinical pressure ulcers often involve polymicrobial infections. Second, while murine models offer practical advantages, they do not fully replicate human wound healing. In addition, our cytokine analysis, based on wound fluid, provides a localized assessment that may not reflect systemic responses.

In conclusion, this model offers a reliable platform for investigating new therapeutic strategies and understanding the mechanisms of bacterial persistence in pressure ulcers. The non-invasive monitoring approach reduces animal usage while providing comprehensive data on infection dynamics and treatment responses. Future studies using this model could explore host-pathogen interactions, bacterial adaptation mechanisms, and novel treatment combinations, ultimately contributing to improved clinical management of pressure ulcer infections.

## Data Availability

The raw data supporting the conclusions of this article will be made available by the authors, without undue reservation.
