# Supplementary figures and images for: A murine pressure ulcer model for evaluating persistence and treatment of Staphylococcus aureus infection
Source: Front Med (Lausanne). 2025 Apr 3;12:1561732. doi: 10.3389/fmed.2025.1561732 (PMC12003373; doi:10.3389/fmed.2025.1561732)

Supplementary Figure 1.

A

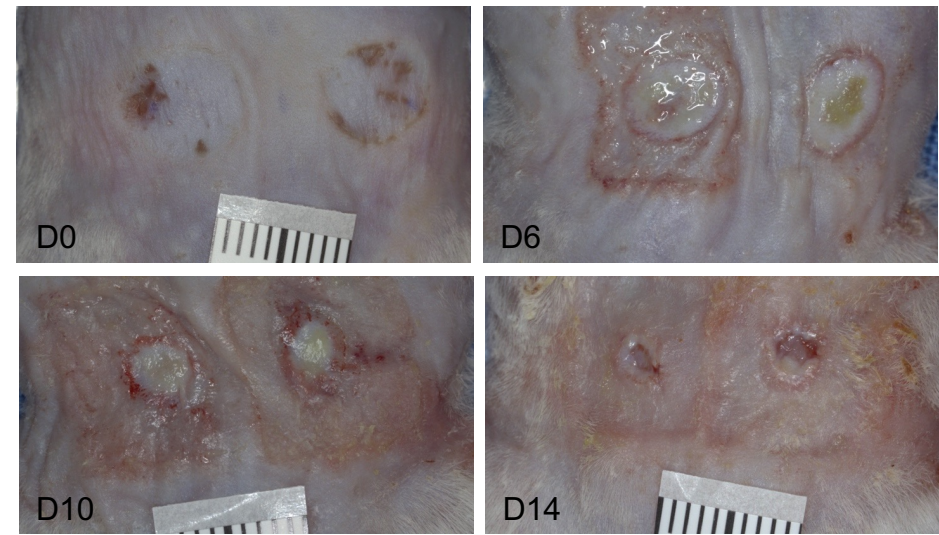

B

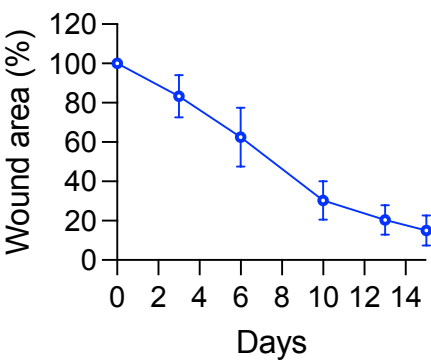

Supplementary Figure 2.

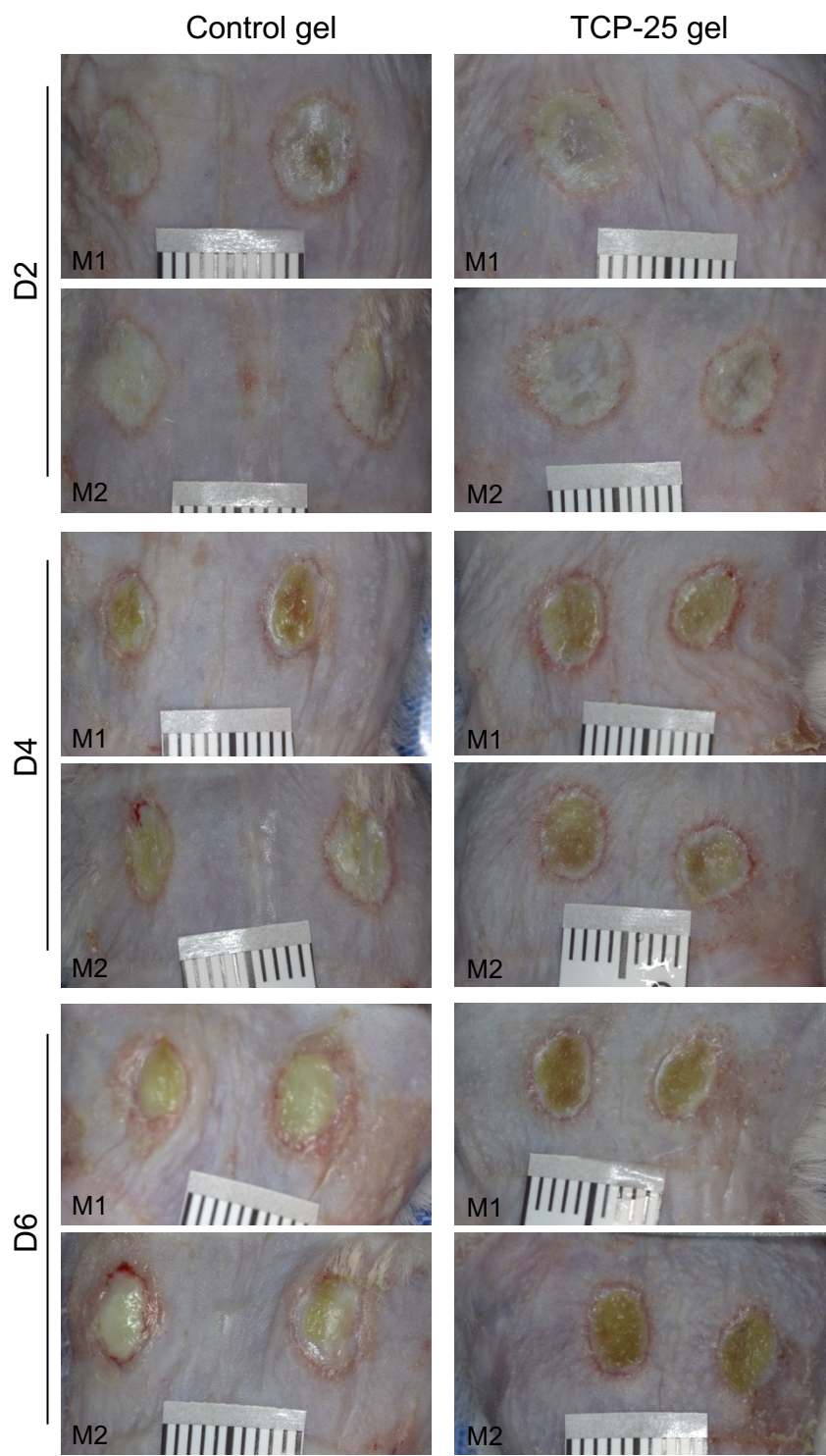

Supplement: Supplementary file 1 [file Data_Sheet_1.PDF]
